# Signs of continental ancestry in urban populations of Peru through autosomal STR loci and mitochondrial DNA typing

**DOI:** 10.1371/journal.pone.0200796

**Published:** 2018-07-18

**Authors:** Francesco Messina, Tullia Di Corcia, Michele Ragazzo, Cesar Sanchez Mellado, Irene Contini, Patrizia Malaspina, Bianca Maria Ciminelli, Olga Rickards, Carla Jodice

**Affiliations:** 1 Department of Biology, University of Rome Tor Vergata, Rome, Italy; 2 Central Laboratory of National DNA Database, Department of Penitentiary Administration, Italian Ministry of Justice, Rome, Italy; 3 Faculty of Intercultural Education and Humanity, National Intercultural University of Amazon, Yarinacocha, Coronel Portillo, Ucayali, Peru; Universita degli Studi di Pavia, ITALY

## Abstract

The human genetic diversity around the world was studied through several high variable genetic markers. In South America the demic consequences of admixture events between Native people, European colonists and African slaves have been displayed by uniparental markers variability. The mitochondrial DNA (mtDNA) has been the most widely used genetic marker for studying American mixed populations, although nuclear markers, such as microsatellite loci (STRs) commonly used in forensic science, showed to be genetically and geographically structured. In this work, we analyzed DNA from buccal swab samples of 296 individuals across Peru: 156 Native Amazons (Ashaninka, Cashibo and Shipibo from Ucayali, Huambiza from Loreto and Moche from Lambayeque) and 140 urban Peruvians from Lima and other 33 urban areas. The aim was to evaluate, through STRs and mtDNA variability, recent migrations in urban Peruvian populations and to gain more information about their continental ancestry. STR data highlighted that most individuals (67%) of the urban Peruvian sample have a strong similarity to the Amazon Native population, whereas 22% have similarity to African populations and only ~1% to European populations. Also the maternally-transmitted mtDNA confirmed the strong Native contribution (~90% of Native American haplogroups) and the lower frequencies of African (~6%) and European (~3%) haplogroups. This study provides a detailed description of the urban Peruvian genetic structure and proposes forensic STRs as a useful tool for studying recent migrations, especially when coupled with mtDNA.

## Introduction

The rapid advancements in genotyping techniques and the growing availability of genetic data in open databases have greatly improved our view of human population structure. Many regions of the human genome can be analyzed to investigate admixture events among populations from different continents, as those associated with the European colonization and the African slave trade in the Americas. New methods for analysis of genome wide SNPs data contributed to determine the continental ancestry in admixed populations from urban Brazilian people, showing their high degree of admixture along with a strong European contribution [1, 2]. In addition, tetranucleotide microsatellite loci (STRs) showed to be geographically more structured than other nuclear markers, with a good power of discrimination on inter-continental scale [3–7].

Many STRs, having an observed heterozygosity >70%, show a high individual discriminating power. Therefore, these markers are widely used in human individual identification for resolving forensic cases [8–10]. Although autosomal STRs of forensic panels show high heterozygosity and low random match probability values, i.e. the probability of obtaining a match between genotypes of two distinct and unrelated individuals, they are also associated to a good capability of ancestry identification [11]. Therefore, these markers can provide valuable information to evaluate nature and extent of transcontinental admixture in South American populations.

The complex historical origin of urban populations in South America was mainly investigated through uniparental and non-recombining genetic markers (mitochondrial DNA and Y chromosome), by means of region-specific haplotypes or haplogroups [12, 13]. Studies on mtDNA composition in Natives from Peru and Ecuador allowed to reconstruct genetic similarity and to clarify early peopling of these areas [14–17]. However, the geographical structuring of mtDNA haplotypes and haplogroups is not able to clearly assign geographical ancestry of individuals as much as thousands autosomal SNPs can do. The mtDNA captures information on the ancestral maternal contribution, but autosomal markers can reveal different scenarios concerning continental origins: i.e. individuals carrying A, B, C and D mtDNA haplogroups, which are predominantly associated to East Asian or Native American ancestry, can turn out to harbor a different ancestry when studied at the level of autosomal markers [18]. Combined analyses of autosomal SNPs and mtDNA data in South American mixed populations have indeed highlighted clear signals of sex-biased genetic inputs from the different continental components [18–20].

**Fig 1:**
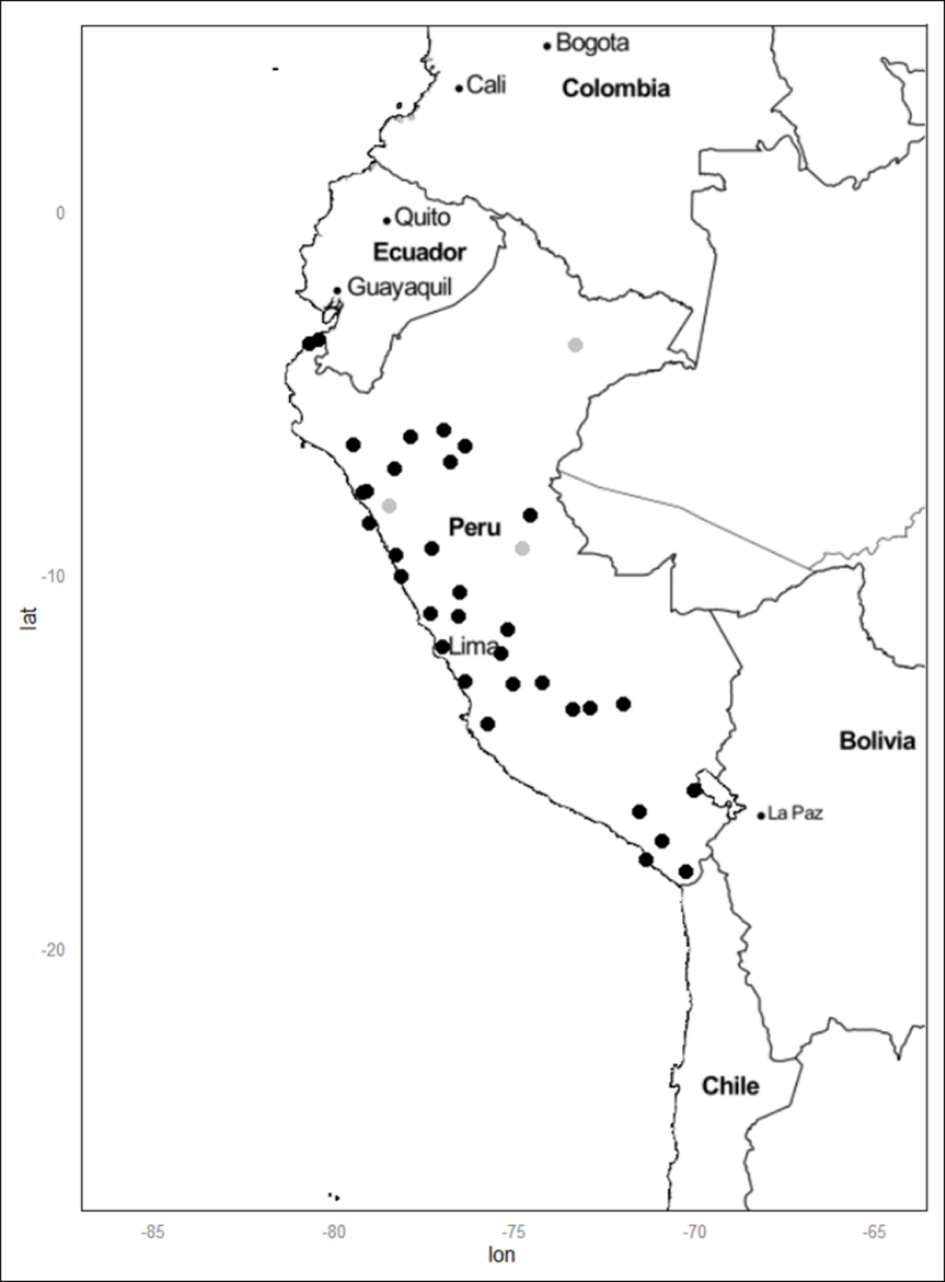
Map of sampling areas. The map shows the birthplace of sampled Peruvian urban and native individuals. Gray dots are native communities, black dots are towns or small cities.

In this work, we analyzed 16 STR loci, commonly used in forensic science, in Native Amazon Peruvians from Ucayali, Loreto and Lambayeque regions and in Peruvians from Lima's urban area and other urban areas of Peru (Fig 1). Moreover, we sequenced the D-loop non-coding region and several SNPs in the coding region of mtDNA to estimate external maternal contributions to the urban Peruvian population. The aim of this work was to quantify, through statistical methods of cluster analysis, the extent of recent migrations in the urban Peruvian population. The results increased our knowledge on Peruvian continental ancestry highlighting effective signs of admixture also in high variable loci of the genome.

## Materials and methods

### Populations

Buccal swabs of a total of 296 individuals were sampled across the entire territory of Peru during three sampling campaigns along the years 2012–2015. Of these, 156 were Native Amazonian Peruvians belonging to Ashaninka, Cashibo and Shipibo people from Ucayali, Huambiza from Loreto and Moche from Lambayeque, while 140 were individuals from the urban area of Lima and other 33 Peruvian towns (S1 Table and Fig 1). The indigenous individuals of the present study were sampled in their own communities settled in the Amazon rainforest or in the desert of Morrope, while urban Peruvian people were sampled in urban areas and the close countryside. The project was also approved by the Ethics Committee of the University of Rome Tor Vergata (June 22nd 2011). Each subject was also asked to report the origin of his/her parents in order to exclude recent immigrants from other continents, and to sign a written informed consent according to the guidelines of the Ethics Committee of University of Rome Tor Vergata. The buccal swab samples were then sent to the Centre of Molecular Anthropology of University of Rome Tor Vergata. The essential information about the samples are given in S1 Table: based on sampling location, for each individual the area of origin (urban area of Lima, North, South and Centre of Peru) and the ecoregion (Rainforest or "Selva", Mountain or "Sierra", Coast or "Costa" and Lima’s urban area) were reported. Sample information on the linguistic group were unknown and samples from urban areas were referred to as "urban", because of the lack of information on ethnicity.

### Laboratory methods

Genomic DNA was extracted using standard procedures [21] and amplified with the commercial kit commonly used for forensic analyses AmpFLSTR^®^ NGM SElect™ PCR Amplification Kit (Applied Biosystems, Foster City, CA) for the D10S1248, vWA, D16S539, D2S1338, Amelogenin, D8S1179, D21S11, D18S51, D22S1045, D19S433, TH01, FGA, D2S441, D3S1358, D1S1656, D12S391 and SE33 loci [22, 23]. After the amplification, all PCR products were separated with the same ABI PRISM 3500 XL Genetic Analyzer, polymer and capillary types, and constant run conditions across the plate set (Life Technologies, Foster City, CA), while the analysis of DNA profiles was carried out using the software GeneMapper^®^ ID-X (Life Technologies, Foster City, CA). All runs included a negative (water) control, 6 replicates of the reference allelic ladder included in the kit, as well as the positive control provided by the manufacturer (Control DNA 007). Profiles were inspected by two independent operators. Independent spreadsheets were produced and compared. Profiles with missing amplification at one or more loci were discarded.

To detect hidden relatedness, we also ran the program Familias 3. 2. 1 [24] using allele frequencies obtained in the whole series. For comparisons, allele frequency databases of US Hispanics [25] and North American Native Americans [26] were employed. Thresholds for the likelihood ratio took into account the number of pairwise comparisons within each population sample [5]. This step led to the exclusion of 64 subjects, since they were identified as Parent/Offspring or Full Sibs (8 urban Peruvians, 5 Ashaninka, 36 Cashibo, 15 Shipibo) (S1 Table) leading to a total sample size of 100 Native Amazon and 132 urban Peruvian individuals.

The mtDNA of 132 urban Peruvian samples and 10 Native Amazon individuals belonging to Moche population were analyzed by sequencing, while mtDNA haplotypes of the other Native individuals were already published [15]. The amplification of the first and second hypervariable segments (HVS-I and HVS-II) of the mtDNA control region was carried out in a 25 μl reaction volume under standard conditions [27]. The primers in the amplification reactions allowed sequences to be read from nucleotide position np 15996 to np 16401 and from np 00029 to np 00408 for HVS-I and HVS-II, respectively [14, 27, 28]. Sequence data were obtained using fluorescent dye labeling and the ABI PRISM 3130 AVANT DNA Sequencer (Applied Biosystems, Foster City, CA) following the manufacturer’s protocols. HVS-I and HVS-II sequences were compared with the revised Cambridge reference sequence [29, 30]. After alignment, control-region haplotypes were analyzed via the HaploGrep website, obtaining phylogenetically classification with a high confidence percentage (>85%) [31]. Moreover, to improve the haplogroup classification, several selected diagnostic SNPs in the mtDNA coding region (8281-8289d, 489C, 493G, 10400T) were assayed by PCR amplification and sequencing [32].

### Statistical analysis on microsatellites and mitochondrial DNA data

Allele frequencies, observed and expected heterozygosity, Fis and Fst values, and the exact test for the Hardy-Weinberg equilibrium (HWE) were calculated using Arlequin v. 3. 5. 2. 2 and 1 million steps in Markov chain [33].

To estimate possible contribution of non-Native American source populations to the urban Peruvian gene pool, we added to our data set genetic profilesfrom two different population samples both from USA [25]: one of European ancestry (US Europeans) and one of African ancestry (US Africans). First, we applied the program STRUCTURE 2. 3. 2 [34] using the admixture model with correlation between allele frequencies. The number of clusters (K) investigated ranged from 2 to 6, and for each K, a burn-in of 50,000 iterations, followed by 50,000 iterations of MCMC (Markov Chain Monte Carlo method) was applied for estimates of clustering.

Principal Component Analysis (PCA), based on individual STR profiles, was carried out by R package *factoextra* to graphically represent affinities among all genotypes and to ascertain which alleles mainly contributed to between-individuals diversity.

To assess the relationships between different possible population sources (US Africans, US Europeans and Native Amazon Peruvians) and urban populations, an independent evaluation of membership probabilities for each individual in each population was obtained by means of Discriminant Analysis of Principal Components (DAPC). This multivariate method defines a model in which the component of genetic variation between groups is maximized by minimizing the within-group component [35]. Analyses were performed using the R package *adegenet* [36]. Then, allele frequencies were submitted to a centered PCA, and the best fitting model in the wide STR database was identified by the function *find*. *cluster*. The retained PCs (100) were passed to a Linear Discriminant Analysis and the first two components were shown on Scatterplots of the DAPC.

For maternal ancestry identification, each mtDNA was phylogenetically classified and standard diversity molecular indices and Tajima's D test of neutrality were calculated for all populations in our database on the basis of the HVS-I haplotype using the software Arlequin v. 3. 5. 2. 2 [33, 37]. Using HVS-I data for each population as output, computation of pairwise genetic Fst matrix and AMOVA was done with Arlequin v. 3. 5. 2. 2 [33, 37] and the significance tested through 10,000 permutations (p<0.05). To represent Fst matrix, a non-metric multidimensional scaling analysis (nmMDS) was performed using PAST version 2. 16 software [38, 39]. The stress values related to the goodness of fit in two-dimensional space yielded results that were acceptable for the plots [38]. The 3D representation of nmMDS was made by R package plot3D, while Mantel test was calculated by Passage 2 software, using 10,000 permutations [40]. Geographic distances in kilometers were calculated on the Great Circle, using appropriate R script, while altitude distances were calculated on Euclidean distance by Passage 2 software.

## Results

### Microsatellite diversity

After relationship filtering, the final dataset comprised 232 subjects (100 Native Amazon and 132 urban Peruvian individuals), all typed at 16 STR loci (S1 Table). The number of alleles per locus varied between 6 (locus D10S1248) and 28 (SE33). Overall, 183 alleles were recorded and the exact test for the Hardy-Weinberg equilibrium (HWE) for all loci did not show departures from the expectation (S2 Table).

To check for a decrease of heterozygosity, Fis indices were calculated for all Native Amazon and urban Peruvian populations. They were quite symmetrical around 0, with no significant values (Table 1). However, it is notable that most of the urban Peruvian samples showed slightly positive Fis values, whereas most of the Amazon samples had slightly negative Fis values. We compared the inbreeding Fis values with those obtained in comparable Native Amazon and mixed American populations typed for 645 STRs [41]. Karitiana, the only Native American population from Brazilian Amazon reported in [41], showed a Fis value of -0.0126079, which was in agreement with excess of heterozygosity in all here studied Amazon samples from Peru. On the other hand, Fis values in admixed populations from Mexico, Brazil, Colombia and Argentina displayed reduced heterozygosity (Fis values >0), as shown also in our urban Peruvian samples.

**Table 1:** Genetic diversity values of STR loci in Native and urban Peruvians.

Population		Original sample size	Final sample size*	Hobs	Hexp	Fis	P value
Ashaninka	Native	19	14	0. 77679	0. 70403	-0. 10774	0. 994455
Cashibo	Native	47	11	0. 625	0. 613	-0. 02049	0. 685446
Huambiza	Native	22	22	0. 72727	0. 71882	-0. 01205	0. 631485
Mochica	Native	10	10	0. 7625	0. 72237	-0. 05882	0. 894356
Shipibo	Native	58	43	0. 70349	0. 71	0. 00928	0. 363663
Lima	Urban	68	63	0. 76389	0. 78295	0. 02454	0. 075347
Coast	Urban	20	20	0. 75937	0. 77989	0. 02698	0. 210495
Mountains	Urban	46	43	0. 79506	0. 77281	-0. 02914	0. 919505
Forest	Urban	6	6	0. 77083	0. 77841	0. 0107	0. 442673

* obtained after Familias 3. 1. 2 filtering.

Hobs, observed heterozygosity; Hexp, expected heterozygosity; Fis, fixation index considering the individual level.

### Continental ancestral information from microsatellite database

We performed an exploratory analysis to highlight genetic structure caused by different continental ancestries. The best clustering model was identified by STRUCTURE 2. 3. 2 software [34], evaluating the maximal value of lnP(D) for each cluster (K) [42]. A 3 K model (lnP(D) = -58263) was chosen as the best clustering model, because all other tested K had lower lnP(D) values. However, we plotted also a 2 K model (lnP(D) = -59135) (S1 Fig). In the 3 K model, the Native Amazon individuals were characterized by only one main component shared with urban Peruvian populations, which was very rare or absent in the two source populations (US Europeans and US Africans). On the other hand, urban Peruvians showed a strong heterogeneity; in fact, on the Native American background an African component was also present, especially in the Lima sample. The strength of the Native component was already evident in the 2 K model.

**Fig 2:**
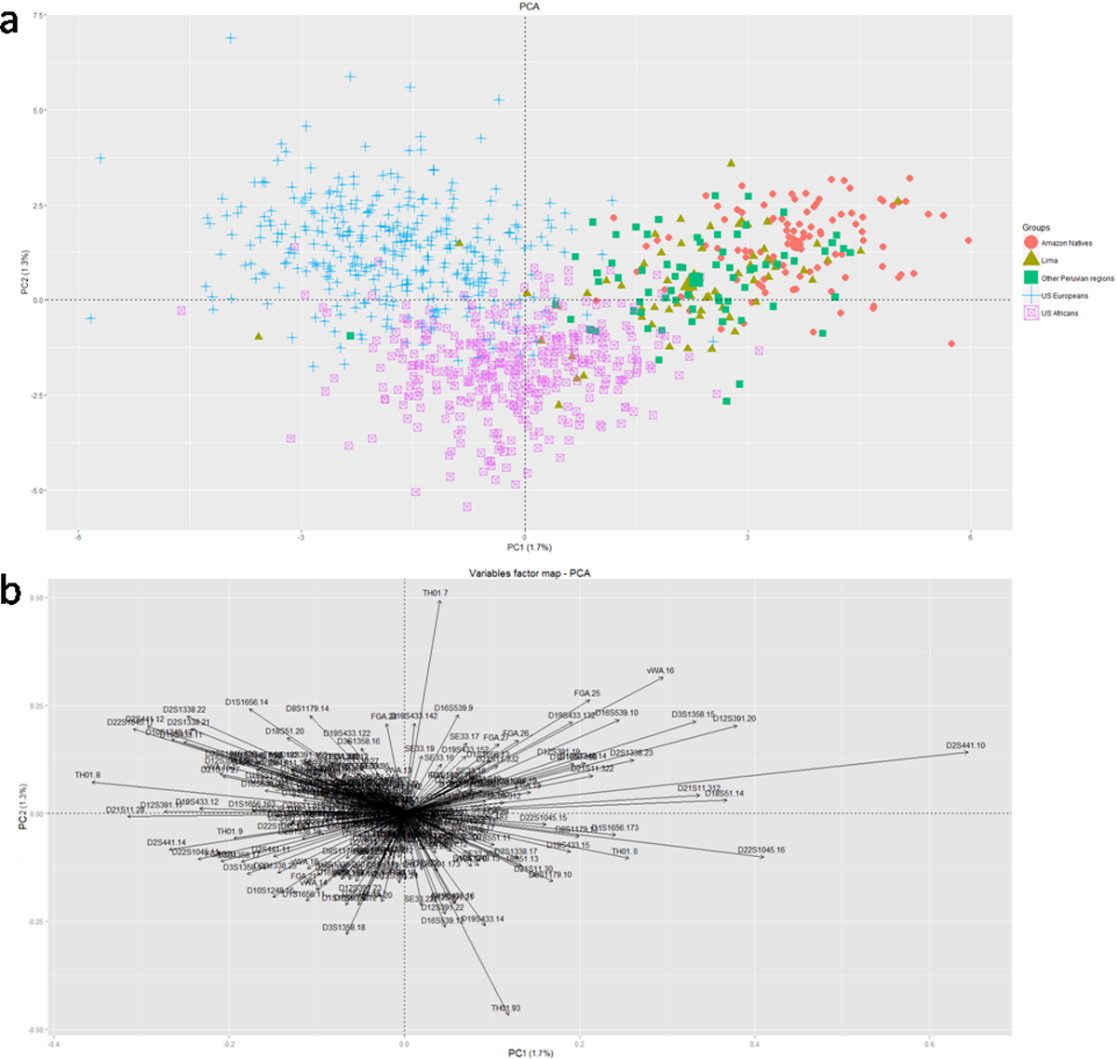
**a) PCA plot based on STR genotypes of urban and Amazon natives Peruvian, US European and US African populations.** Dots represent individuals and the colors are associated with geographic origin. The first principal component accounts for 1. 7% of the total variability, while the second principal component accounts for 1. 3%. **b) Contributions of each STR allele to PCA plot**. The main allele contributors to the first and second PCs are shown.

PCA based on STR genotypes mainly confirmed admixed structure of urban Peruvians contributed by Native Amazon and African populations (Fig 2A). The total variance percentage of PC1 and PC2, was 3% (PC1 1.7% and PC2 1.3%). The position of both Native Amazon people and urban Peruvians was sharply influenced by the contribution of the D2S441-10 allele, the most frequent in our populations (Native Amazon 0.56439 and urban Peruvians 0.675) (Fig 2B). The overlapping centroids for Lima and other Peruvian urban regions suggested the same degree of admixture.

A comparable degree of admixture for Lima and other Peruvian urban regions was confirmed not only by a null Fst value (-0.00138; not significant) of Lima vs. pooled data of the other Peruvian regions, but also by null Fst values between the single population samples (S3A Table). Considering whole dataset as only 4 populations (Native Amazon, Urban Peruvians, US Europeans and US Africans), the lowest Fst value was obviously observed between urban Peruvians vs. Native Amazon (Fst = 0. 0144; p = 0. 000). The Fst urban Peruvians vs. US Africans (Fst = 0. 0276; p = 0. 000) was lower than that urban Peruvians vs. US Europeans (Fst = 0. 0368; p = 0. 000),thus allowing us to further confirm the African contribution in urban Peruvians (S3B Table). Table 2 reports Fst values for each STR locus, calculated both for Native Amazon vs urban Peruvian populations and for Native Amazons, urban Peruvians, US Europeans and US Africans.

**Table 2:** Inter population diversity fixation index (Fst) values for each STR locus, calculated both for Native Amazon *vs* urban Peruvian populations and for Native Amazons, urban Peruvians, US Europeans and US Africans.

	Native Amazons *vs* urban Peruvians		Native Amazons, urban Peruvians, US Europeans and US Africans	
Locus	Fst	P values	Fst	P values
D10S1248	0. 03915	0. 0014	0. 02842	0. 0000
vWA	0. 01645	0. 1681	0. 02987	0. 0000
D16S539	0. 01487	0. 1929	0. 02628	0. 0000
D2S1338	0. 03758	0. 0000	0. 03001	0. 0000
D8S1179	0. 0018	0. 9765	0. 01500	0. 0000
D21S11	0. 00287	0. 9683	0. 02703	0. 0000
D18S51	0. 02031	0. 0163	0. 02606	0. 0000
D22S1045	0. 06212	0. 0000	0. 05902	0. 0000
D19S433	0. 02703	0. 0026	0. 02828	0. 0000
TH01	0. 02336	0. 0320	0. 06916	0. 0000
FGA	0. 03497	0. 0000	0. 03134	0. 0000
D2S441	0. 02307	0. 0433	0. 10050	0. 0000
D3S1358	0. 01187	0. 3849	0. 03775	0. 0000
D1S1656	0. 02333	0. 0026	0. 02846	0. 0000
D12S391	0. 01493	0. 1675	0. 03516	0. 0000
SE33	0. 01657	0. 0282	0. 01132	0. 0000
Mean	0. 02261		0. 03579	

**Fig 3:**
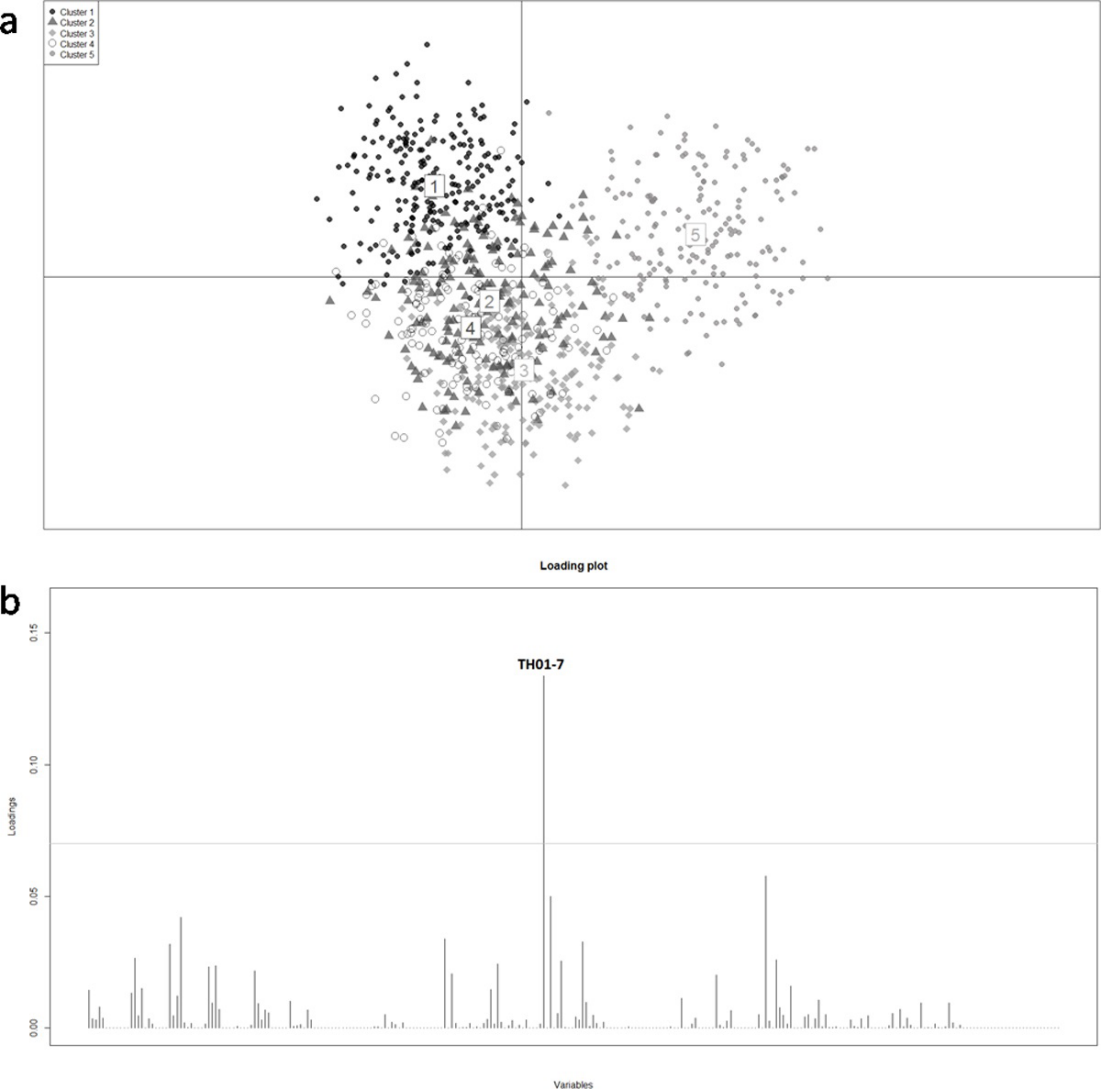
**a) DAPC of STR genotype database.** DAPC of STR genotype database of urban and Amazon native Peruvian, US European and US African populations. Scatter plot showing the first two principal components. Dots represent individuals. **b) Loading plot of DAPC.** The main allele contributors to individual DAPC clustering are shown.

We used DAPC to define clusters of genetically related individuals. The best fitting model in the wide STR database was 5 K (BIC = 1755.36). After a Linear Discriminant Analysis, the first two components were represented on Scatterplots of the DAPC (Fig 3A), which showed the same trend of the STR genotypes PCA. Clusters 1 and 5 were strongly defined and located respectively in first and second quarters, while clusters 2, 3 and 4 resulted widely overlapping and undistinguished. The TH01-7 allele was underlined as the main contributor to individual clustering, posing threshold 0.07 loadings (TH01-7 = 0. 13380305 loading value) (Fig 3B). The height of each bar is proportional to the contribution of each allele (loading). When threshold loading was set to 0.05, also D1S1656-14 exceed it (loading value = 0.05763481). The strong contribution of the TH01-7 allele was not a surprise: the amount of genetic diversity, preserved in the TH01 locus, was described by high Fst value (Fst TH01 entire STR dataset = 0.06916; Table 2). The TH01-7 allele showed high frequencies in both urban (33% - 45%) and Amazon (35% - 68%) populations. In US Europeans its frequency was 19%, while in the US Africans was 40%. The strongly different allele frequencies in the dataset contributed to the scattered distribution of genotypes on the plot.

**Fig 4:**
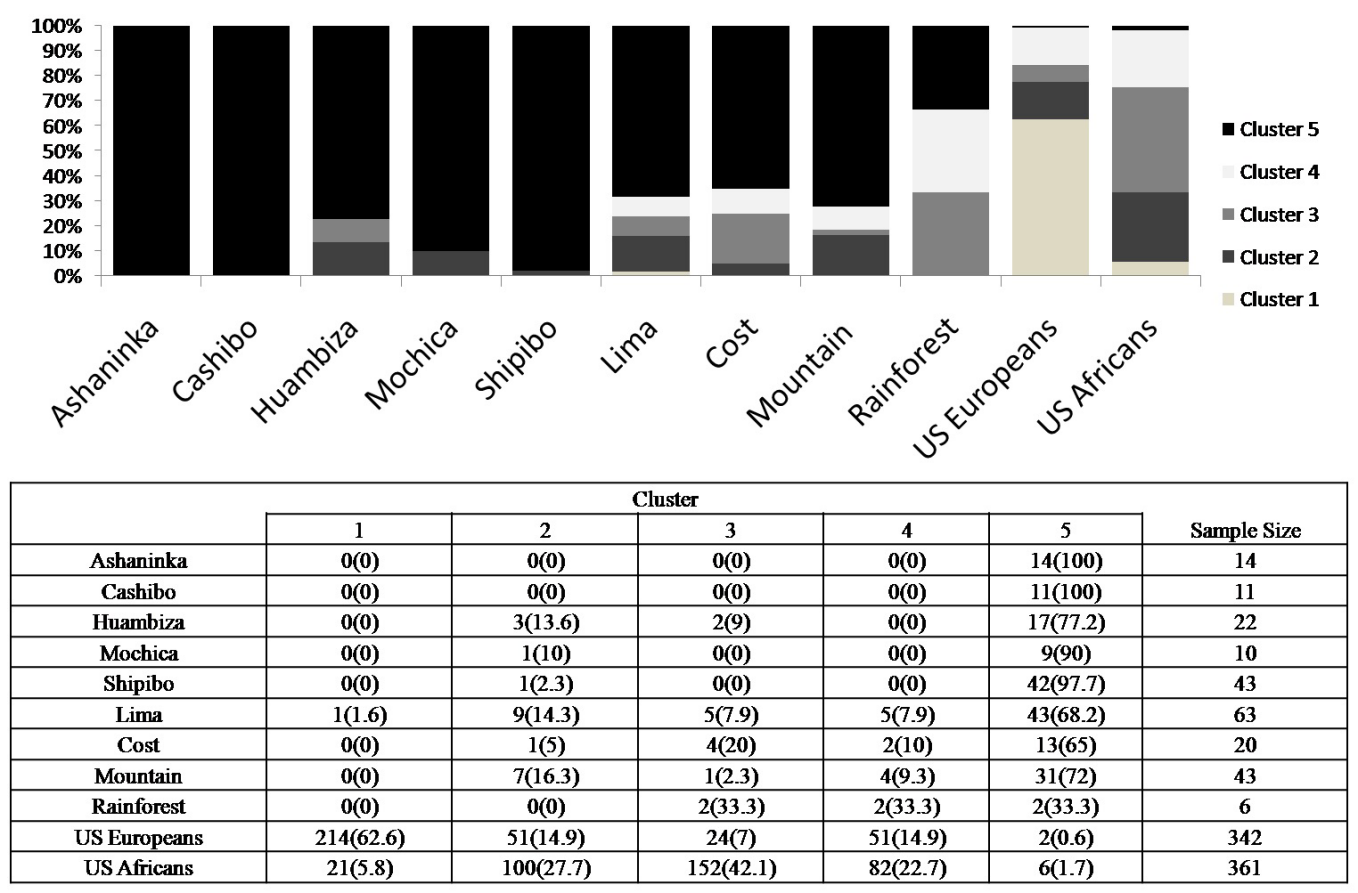
Composition of DAPC clusters for STR genotypes. Vertical bars represent the proportion (%) of each cluster in each population. In the table the absolute and relative (%) frequencies of each cluster for each population are reported.

The model highlighted an association between some clusters and the populations under study (Fig 4). Specifically, Native Amazon individuals were found typically within cluster 5, US Africans within clusters 2 and 3, while US Europeans in cluster 1. Cluster 4 seems not to be associated with specific populations. Cluster 5 contains most Native Amazon (77.2% - 100%) and urban Peruvian individuals (33.3% - 72%), while individuals of the source populations were almost absent. Clusters 2 and 3 clearly marked individuals belonging to the US African sample (27% for cluster 2 and 42.1% for cluster 3). It is worth noticing that many urban and few Native Peruvian individuals fall into African clusters 2 and 3. Instead, cluster 1 is almost exclusive of US Europeans, and only one individual from Lima was found in this cluster. At last, the origin of cluster 4 remained unknown and probably it could be attributed to mixed individuals between source populations.

### Mitochondrial genetic diversity

The results of clustering of STR profiles obtained by DAPC were compared with those from mtDNA analysis. mtDNAs of 132 urban Peruvian and 10 Moche individuals were newly genotyped, while the haplotypes of the remaining 90 Native Amazons were already available [15]. In S1 Table, for each subject, the variants of mtDNA HVS-I, HVS-II and coding regions are listed, along with the haplogroup and STR cluster affiliations.

Table 3 reports the haplogroup frequencies for the urban Peruvian sample. Most of the mtDNA haplogroups were of Native American origin (6. 1% A, 51.5% B, 15.2% C, 17.4% D), while 3.2%, 7.1% and 0.8% were of European, African and Asian origin, respectively.

**Table 3:** Absolute and relative (%) frequencies of mtDNA haplogroups in urban Peruvian sample.

	mtDNA haplogroup frequencies
A2	8 (6.1)
B2	24(18.2)
B4	44(33.3)
C1	20(15.2)
D1	18(13.6)
D4	5(3.8)
H	1(0.8)
HV0	1(0.8)
I5a2	1(0.8)
L1	2(1.5)
L2	5(3.8)
M1a1i	1(0.8)
M7c	1(0.8)
W3a1	1(0.8)

To accurately estimate the native contribution, a dataset reporting only mtDNA haplotypes belonging to the Native American haplogroups A, B, C and D was created for urban Peruvian sample, and it was then compared with the mtDNA Native haplotype dataset of admixed and Native people from Peru, Bolivia, Chile and Amazon region of Brazil (Table 4). The genetic diversity parameters in urban Peruvian samples did not differ from those of other South American populations. All Tajima’s D values were negative indicating no selection on mtDNA, nevertheless, after applying Bonferroni correction (p<0. 004),only two p values were significant (Table 4). In Table 3 the haplogroup frequencies for urban Peruvian sample are reported. Most of the mtDNA haplogroups were of Native American origin (6.1% A, 51.5% B, 15.2% C, 17.4% D), but European (3.2%), African (7.1%) and Asian (0.8%) matrilineal inputs were also found.

**Table 4:** Diversity indices of mtDNA sequences from South America belonging to Native American haplogroups A, B, C and D.

Samples	Population	N	K	S	H	MSPD	π	Tajima's D	Tajima's D p value	References
Urban Peru	Urban	119	70	67	0. 9708+/-0. 0074	6. 298533+/-3. 0092	0. 015707+/-0. 0083	-1. 57923	0. 02	Thispaper
North and Central Andes Peru	Natives	99	64	60	0. 9862+/-0. 0040	6. 135848+/-2. 9431	0. 015301+/-0. 0081	-1. 51732	0. 04	[48]; this paper
Amazon Natives Peru	Natives	353	100	84	0. 9699+/-0. 0031	6. 876980+/-3. 2439	0. 017150+/-0. 0089	-1. 39077	0. 04	[15, 16]
La Paz Bolivia	Urban	152	82	69	0. 9656+/-0. 0076	4. 615023+/-2. 2775	0. 011509+/-0. 0063	-1. 94672	0. 003	[61]
Llandos Bolivia	Natives	171	99	88	0. 9826+/-0. 0039	7. 511730+/-3. 5252	0. 018732+/-0. 0097	-1. 59691	0. 02	[61]
Sub Andes Bolivia	Urban	127	81	70	0. 9696+/-0. 0086	4. 763280+/-2. 3442	0. 011879+/-0. 0065	-1. 99901	0. 002	[61]
Titicaca Peru	Natives	132	83	65	0. 9865+/-0. 0035	5. 567083+/-2. 6913	0. 013883+/-0. 0074	-1. 67358	0. 02	[17, 48]
Amazon Brazil	Natives	237	61	47	0. 9594+/-0. 0046	5. 093685+/-2. 4797	0. 012702+/-0. 0068	-1. 00604	0. 16	[65]
Temuco Chile	Urban	69	36	46	0. 9633+/-0. 0104	5. 495311+/-2. 6757	0. 013704+/-0. 0074	-1. 40415	0. 06	[62]
Santiago de Chile	Urban	167	85	70	0. 9685+/-0. 0073	6. 831325+/-3. 2327	0. 017036+/-0. 0089	-1. 37281	0. 05	[62]
Punta Arenas Chile	Urban	78	32	42	0. 9194+/-0. 0210	5. 939061+/-2. 8645	0. 014811+/-0. 0079	-0. 97837	0. 16	[62]
Iquique Chile	Urban	189	90	80	0. 9749+/-0. 0050	6. 902961+/-3. 2617	0. 017214+/-0. 0090	-1. 53332	0. 03	[62]
Conception Chile	Urban	178	74	62	0. 9683+/-0. 0053	6. 670729+/-3. 1626	0. 016635+/-0. 0087	-1. 16074	0. 1	[62]
Natives South Argentina-Chile	Natives	204	75	62	0. 9451 +/-0. 0112	6. 378779 +/-3. 0351	0. 015907+/-0. 0084	-1. 18661	0. 1	[66, 67]

Sample size (N), number of haplotypes (K), number of polymorphic sites (S), haplotype diversity (H), mean number of pairwise differences (MSPD), nucleotide diversity (π) and Tajima's D test.

Only 13 mtDNAs (10%) out of the 132 genotyped in urban Peruvians belonged to non-Native American haplogroups (Table 5): 8 belonged to Sub-Saharan haplogroups (6%), 4 to European haplogroups (3%) and 1 to an Asian haplogroup (1%). With the exception of two mtDNAs belonging to the African haplogroup L2a1 found in the Lima population, all the others differed from each other. Among the 13 non-Native mtDNAs, 7 were carried by individuals belonging to STR cluster 5 (Native American), the remaining 6 (5% of the overall mtDNA dataset) belonged to individuals of non-Native STR clusters, but none of them was associated to STR cluster 1 (only European) (Table 5). Moreover, all these 13 mtDNAs were from the Lima’s urban area and other urban Coast regions (S2 Fig), suggesting a sex-biased geographical distribution of admixture events.

**Table 5:** mtDNAs from urban Peruvian samplesbelonging to non-native mtDNA haplogroups.

Sampling place	Region	Geographic position	Natural region	Longitudine	Latitudine	Altitudine	Cluster DAPC	mtDNA haplogroup	Origin haplogroup	HVS-I and HVS-II sequence variation
Lima	Lima	Central	Urban Area	-77. 014	-12. 001	149 m	04	L2b'c	African	73G 146C 150T 152C 195C 198T 263G 16223T 16270T 16278T 16390A
Lima	Lima	Central	Urban Area	-77. 014	-12. 001	149 m	03	L2a1	African	73G 146C 152C 195C 199C 263G 16223T 16278T 16294T 16309G 16390A
Lima	Lima	Central	Urban Area	-77. 014	-12. 001	149 m	05	L1c2b	African	73G 151T 152C 182T 186A 189C 195C 198T 16093C 16129A 16187T 16189C 16223T 16265C 16278T 16286G 16294T 16311C 16360T
Lima	Lima	Central	Urban Area	-77. 014	-12. 001	149 m	05	L2a1c2	African	73G 143A 146C 152C 195C 263G 16213A 16223T 16278T 16294T 16309G 16390A
Lima	Lima	Central	Urban Area	-77. 014	-12. 001	149 m	02	HV0	European	72C 263G 16082T 16097C 16298C
Lima	Lima	Central	Urban Area	-77. 014	-12. 001	149 m	05	L2a1	African	73G 146C 152C 195C 199C 263G 16223T 16278T 16294T 16309G 16390A
Lima	Lima	Central	Urban Area	-77. 014	-12. 001	149 m	02	W3a1	European	73G 189G 194T 199C 204C 207A 263G 16223T 16292T
Lima	Lima	Central	Urban Area	-77. 014	-12. 001	149 m	05	H	European	263G
Chiclayo	Lambayeque	North	Coast (Costa)	-79. 482	-6. 4353	30 m	04	L2a	African	73G 143A 146C 152C 195C 263G 16223T 16278T 16294T 16309G 16390A
Chiclayo	Lambayeque	North	Coast (Costa)	-79. 482	-6. 4353	30 m	04	M1a1i	African	73G 195C 204C 16129A 16182C 16183C 16189C 16223T 16249C 16311C 16359C
Tumbes	Tumbes	North	Coast (Costa)	-80. 427	-3. 5564	26 m	05	L1c3c	African	73G 93G 151T 152C 182T 186A 189C 195C 16129A 16187T 16189C 16223T 16278T 16293G 16294T 16311C 16360T
Arequipa	Arequipa	South	Mountain (Sierra)	-71. 537	-16. 409	2320 m	05	I5a2	European	73G 199C 250C 263G 16086C 16129A 16148T 16223T 16391A
Puno	Puno	South	Mountain (Sierra)	-70. 021	-15. 840	3819 m	05	M7c	Asian	73G 143A 146C 263G 16223T

Pairwise genetic Fst matrix was built on the mtDNA HVS-I haplotype data obtained from the present research and other populations from urban, Amazon and Andean places of the South American West Coast. They were plotted through 3D nmMDS to identify maternal genetic relationships with 0.02 Stress value (S3 Fig). The lines under each point highlight the distance on Third Dimension, while different colors (black-to-red) help to visualize the Second Dimension. This plot showed four main population groups: "Amazon" group (including AmazonPeru, AmazonBrazil and LlandosBolivia), "Lake Titicaca" group (LaPazBolivia, SubAndesBolivia, TiticacaPeru and also TemucoChile), "Andes Peru" group (NCAndePeru and UrbanPeru) and "Chile" group (NativesSArgentinaChile, SantiagoChile, IquiqueChile, ConceptionChileand Punta Arenas near Tierra del Fuego).

The same populations were grouped according to a geopolitical or ecoregional criterion and for each grouping we performed AMOVA. Grouping described by 3D nmMDS showed greater amount of variance among groups than geopolitical and ecoregional grouping (Table 6). Moreover, the Native mtDNA component of the Temuco sample seems to have a contribution from Lake Titicaca group, as showed also by Fst values between Temuco and La Paz, Bolivian, sub Andes and Lake Titicaca Peru (0.03468, 0.03608 and 0.03929, respectively).

**Table 6:** Percentage of molecular variance amount among groups, among populations within groups and within populations.

Grouping based on	Variance among groups Fct	Variance among populations within groups Fst	Variance within populations Fsc
Geopolitics	5. 51	5. 56	88. 93
Ecoregion	7. 76	4. 11	88. 13
3D nmMDS	9. 45	2. 42	88. 13

Finally, to test possible associations between geography and genetics, the Fst matrix was correlated with both altitudinal and geographical distance matrices among populations. This test showed a light correlation index between altitude and genetics (r = 0.31285, p = 0.014 by Mantel test), while no correlation between genetics and geographical distances (r = 0.102, not significant) was found.

The three different grouping are as follows: Geopolitics (first group: urban Peru, North and Central Andes Peru, Titicaca Peru, Amazon Natives Peru;second group: sub Andes Bolivia, La Paz Bolivia, Llandos Bolivia; third group: Conception Chile,Iquique Chile, Punta Arenas Chile,Santiago de Chile,Natives South Argentina Chile, Temuco Chile; fourth group: Amazon Natives Brazil); Ecoregions (first group: urban Peru, North and Central Andes Peru,sub Andes Bolivia, La Paz Bolivia, Titicaca Peru; second group: Llandos Bolivia, Amazon Natives Peru, Amazon Natives Brazil; third group: Conception Chile, Iquique Chile, Punta Arenas Chile, Santiago de Chile,Natives South Argentina Chile, (Temuco Chile); 3D nmMDS first group (Andes Peru): urban Peru, North and Central Andes Peru;second group (Lake Titicaca): sub Andes Bolivia, La PazBolivia, Titicaca Peru, Temuco Chile; third group (Amazon): Llandos Bolivia, Amazon Natives Peru, Amazon Natives Brazil; fourth group (Chile): Conception Chile, Iquique Chile, Punta Arenas Chile,Santiago de Chile,Natives South Argentina Chile).

## Discussion

In this work, we tried to shed light on the transcontinental contributions to the gene pool of admixed urban Peruvian populations, using recently developed multivariate methods for clustering analysis on STR loci commonly used in individual identification. Moreover, we also took advantage of the geographic origin information provided by the maternally-transmitted mtDNA.

The slightly reduced heterozygosity (slightly positive not significant Fis values) showed by urban Peruvians, may be due to a low level of endogamy in these populations. Inbreeding of urban people in Peru was also confirmed [43] by positive Fis values based on different STR loci of urban populations: Chiclayo, Lima, Piura and Huancayo showed Fis positive values (0.012, 0.010, 0.007 and 0.015, respectively). Similar trends were described for the STR gene pools of Peruvians and other admixed South American populations also in [6].

The clustering and multivariate methods applied on STR genotype database allowed to highlight admixed origin of urban Peruvian populations, in which the African component was evident on the most abundant Native background, especially in the Lima sample (Figs 2A and S1). These findings are in contrast with the STR genotype dataset of admixed populations from the rest of South America, in which a large European component and a considerable Native American component, followed by a small and residual African contribution, seem to constitute a genetic leitmotiv. Such a structure was commonly described in admixed urban populations from Venezuela, Colombia, Brazil and US Hispanics [44, 45]. In urban admixed populations from all over South America, commonly called "Mestizos", typing of autosomal and X chromosome STR loci showed variable Native contribution, ranging from 70% in Andean regions and Meso-America to 20% in Colombia and Central America, while European ancestry resulted the highest external component (from 25% in Chilean Andean region to 70% in Southern Brazilian people). African ancestry in the entire dataset is low (<10%) [46].

As regarding Peru, the genomic ancestry proportions based on autosomal STRs showed 30% of admixture with non-Native American populations [47], while proportions provided by INDEL polymorphisms in Peruvians from Coast, Andes and Amazon were identified as 83% Native American and 17% non-autochthonous, mainly from Europe [48]. These proportions allowed us to consider the results obtained by DAPC reliable (Fig 4). In fact, very many urban Peruvian individuals belonged to the Native cluster (33.3% - 72%), that is the cluster made up of a high percentage of Native Amazon individuals (77.2% - 100%). The low number of individuals in African and European clusters (clusters 2 and 3, and cluster 1, respectively) was strongly consistent with the history of other populations from this part of South America. Moreover, in autosomal SNPs, mtDNA and Y chromosome of Bolivian admixed people, the continental ancestry of Native Americans was the most abundant, followed by European and African ones [49, 50].

The identification of STR alleles with geographic variation on global scale was the other main point of this work. In the present study, the main contributor to individual clustering provided by DAPC was the TH01-7 allele (Fig 3B). High frequencies of this allele, similar to those here observed, were already described in the Andean and coastal population from Peru (43% - 51%) and Native Amazon people from Ecuador (40%) [3, 43]. In Afro-Caribbean people and in African ancestry Colombians this allele showed a 40% frequency [51–54], while in all other South American populations (Brazil, Argentina and Chile) it ranged between 24% and 26%, consistent with European-Native American admixture [55–59]. However, a strong diversity of TH01 allele frequencies on geographic scale was already well known. As described in previous works, the TH01-6 allele showed an increasing West-East cline in Europe, whereas the TH01-9.3 allele displayed a marked latitudinal gradient with high frequencies in Northern Europe [4, 60]. This wide diversity of TH01 allele frequencies could be due to selection or demic events.

The second part of our study extends this discussion through the study of mtDNA background. mtDNA haplotypes belonging to non-Native haplogroups were concentrated only in Lima and on the urban Coast region. In particular, in the here studied urban Peruvians, the African maternal contribution (6%) was slightly more represented than the European maternal contribution (3%) (S2 Fig and Table 5). Indeed, the African component was higher (6%) than in Bolivian and Chilean populations (~1%) [61, 62], whereas the European component (3%) is comparable with that reported in Bolivia (~1%), and less than that found in Chile (~11%), which was strongly involved in a recent migration from Europe [62]. Furthermore, no maternal Old World contributions were identified in Ecuadorians [14, 63].

These data fitted with STR cluster proportions: 13% and 9% of urban Peruvian samples resulted to belong to African clusters 2 and 3, whereas only 1% belonged to the European cluster. These data could suggest past slavery, which especially involved Lima and towns on the Coast region and influenced heavily the population composition of this area: by XVIII century more than a third of the Lima's population included slaves, mainly Africans [64]. These results demonstrate that it is possible to detect signs of admixture events in autosomal STR and mtDNA gene pool on population scale [11, 18].

Finally, the Native mitochondrial component showed a strong similarity in urban Peruvian and Andean populations, indicating Andean people as the most probable Native source population of urban Peruvians. This scenario is plausible, because, unlike Native Amazon populations, Andeans maintained larger population sizes also after European colonization and greater mobility [16]. The analysis of Native mtDNA gene pool revealed that the diversity of the urban Peruvian sample is an integral part of South America mtDNA variability.

## Conclusion

In this work, we tried to shed light on the presumed admixed origin of urban Peruvian populations through clustering and multivariate methods. In the STR genotype database strong signs of continental ancestry were highlighted, also supported by mtDNA composition. Finally, this work confirmed the important role of autosomal STRs and mtDNA for historical reconstructions, underlining the advantage of a combined use of the autosomal and uniparental markers usually employed in forensic applications.

## Supporting information

S1 Fig STRUCTURE analysis at Ks 2 and 3 for urban and Native Amazon Peruvian, US European and US African samples. The colors are as follows: dark grey for European, grey for African and light grey for Native Amazon ancestry component. The presence of more than one component in US European and US African samples was due to the multiethnic origin of United States populations.
(TIF)

S2 Fig Geographic distribution of haplotypes. Geographic distribution of haplotypes,reported in Table 3, belonging to Non-Native mtDNA haplogroups. Color dots were associated with continental origin (red Africa; black Europe; yellow East Asia).
(TIF)

S3 Fig 3D nmMDS of pairwise Fst matrix. 3DnmMDS on the first three axes based on the matrix of pairwise Fst values of HVS-I mtDNA after grouping into 14 geographic samples. Color shades from bright red to black refer to position on dimension 2. The references of all samples were reported: UrbanPeru (this paper); NCAndePeru [48] (this paper); AmazonPeru [15, 17]; LaPazBolivia [61]; LlandosBolivia [61]; SubAndeBolivia [61]; TiticacaPeru [16, 48]; AmazonBrazil [65]; TemucoChile [62]; SantiagoChile [62], PuntaArenas [62], IquiqueChile [62], ConceptionChile [62], NativesSArgentinaChile [66, 67].
(TIF)

S1 Table List of sampled individuals. List of sampled individuals with birthplace and geographic information, geographic coordinates, response after filtering with Familias 3 software, STR DAPC cluster, mtDNA haplotypes and haplogroups.
(XLSX)

S2 Table Table of relative allele frequencies at 16 STR loci. Relative allele frequencies at 16 STR loci Relative allele frequencies at 16 STR loci in the population samples and two pooled samples (only Amazon and urban Peruvians).
(XLSX)

S3 Table Pairwise Fixation Indices (Fst) in all STR loci. Pairwise Fixation Indices (Fst) in all STR loci: a) using all single population samples. b) considering whole dataset as only 4 populations. All values were significant (p < 0.05). Above diagonal P-values; below diagonal pairwise Fst value. In bold: not significant P values.
(XLSX)
