# Supplementary figures and images for: Signs of continental ancestry in urban populations of Peru through autosomal STR loci and mitochondrial DNA typing
Source: PLoS One. 2018 Jul 18;13(7):e0200796. doi: 10.1371/journal.pone.0200796 (PMC6051651; doi:10.1371/journal.pone.0200796)

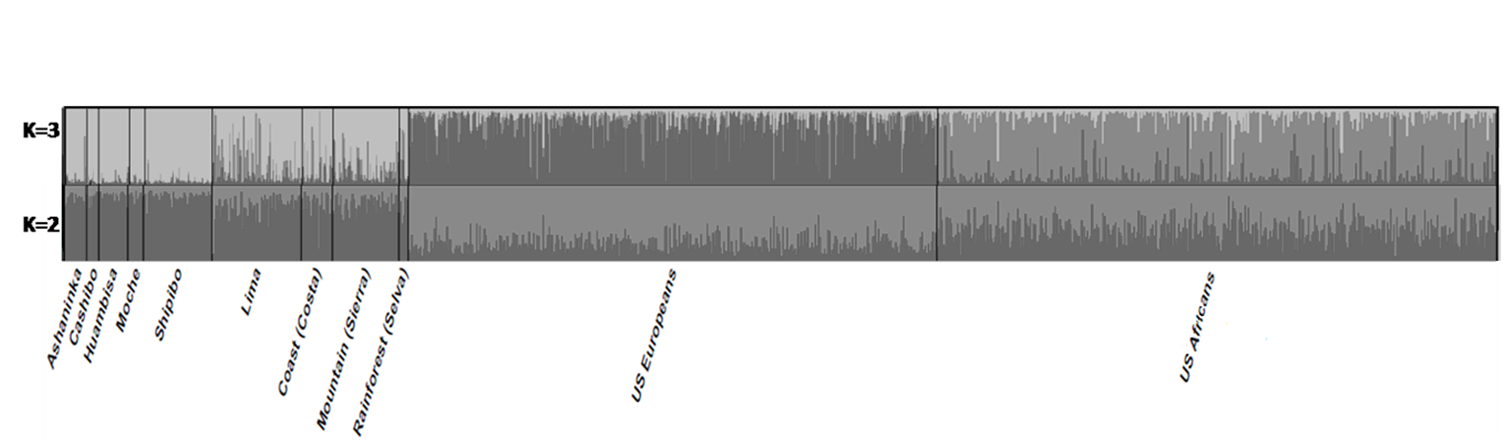

Supplement: S1 Fig — The colors are as follows: dark grey for European, grey for African and light grey for Native Amazon ancestry component. The presence of more than one component in US European and US African samples was due to the multiethnic origin of United States populations. (TIF) [file pone.0200796.s001.tif]

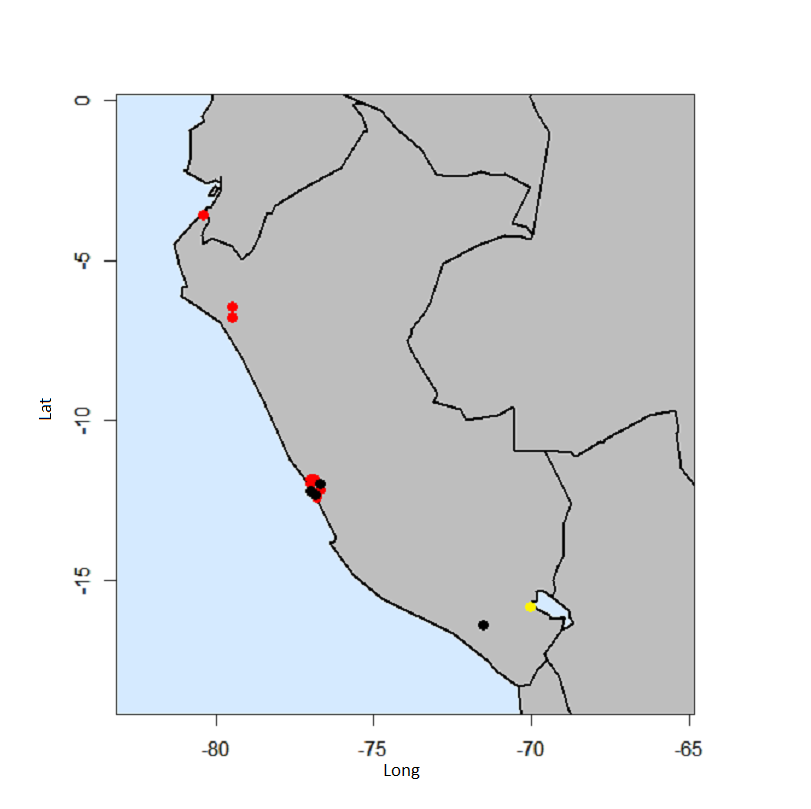

Supplement: S2 Fig — Geographic distribution of haplotypes,reported in Table 3, belonging to Non-Native mtDNA haplogroups. Color dots were associated with continental origin (red Africa; black Europe; yellow East Asia). (TIF) [file pone.0200796.s002.tif]

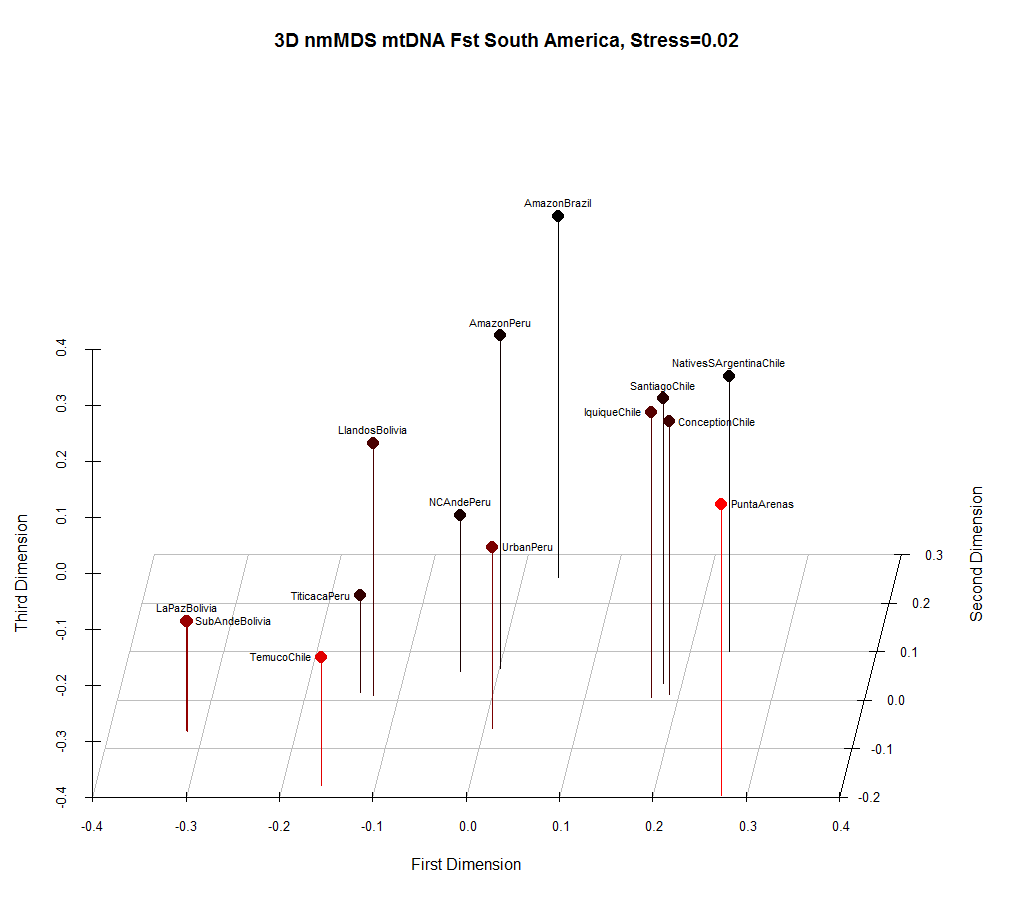

Supplement: S3 Fig — 3DnmMDS on the first three axes based on the matrix of pairwise Fst values of HVS-I mtDNA after grouping into 14 geographic samples. Color shades from bright red to black refer to position on dimension 2. The references of all samples were reported: UrbanPeru (this paper); NCAndePeru [48] (this paper); AmazonPeru [15, 17]; LaPazBolivia [61]; LlandosBolivia [61]; SubAndeBolivia [61]; TiticacaPeru [16, 48]; AmazonBrazil [65]; TemucoChile [62]; SantiagoChile [62], PuntaArenas [62], IquiqueChile [62], ConceptionChile [62], NativesSArgentinaChile [66, 67]. (TIF) [file pone.0200796.s003.tif]
